# Sexual recombination and increased mutation rate expedite evolution of *Escherichia coli* in varied fitness landscapes

**DOI:** 10.1038/s41467-017-02323-4

**Published:** 2017-12-13

**Authors:** George L. Peabody V, Hao Li, Katy C. Kao

**Affiliations:** Department of Chemical Engineering, Texas A&M University, 3122 TAMU, College Station, TX 77843-3122 USA

## Abstract

Sexual recombination and mutation rate are theorized to play different roles in adaptive evolution depending on the fitness landscape; however, direct experimental support is limited. Here we examine how these factors affect the rate of adaptation utilizing a “genderless” strain of *Escherichia coli* capable of continuous in situ sexual recombination. The results show that the populations with increased mutation rate, and capable of sexual recombination, outperform all the other populations. We further characterize two sexual and two asexual populations with increased mutation rate and observe maintenance of beneficial mutations in the sexual populations through mutational sweeps. Furthermore, we experimentally identify the molecular signature of a mating event within the sexual population that combines two beneficial mutations to generate a fitter progeny; this evidence suggests that the recombination event partially alleviates clonal interference. We present additional data suggesting that stochasticity plays an important role in the combinations of mutations observed.

## Abstract (web summary)

Sexual recombination and mutation rate may play different roles in adaptive evolution depending on the fitness landscape. Here, Peabody et al. examine how the two factors affect the rate of adaptation of an *E. coli* strain capable of sexual recombination, under different conditions during experimental evolution.

## Introduction

**Fig. 1:**
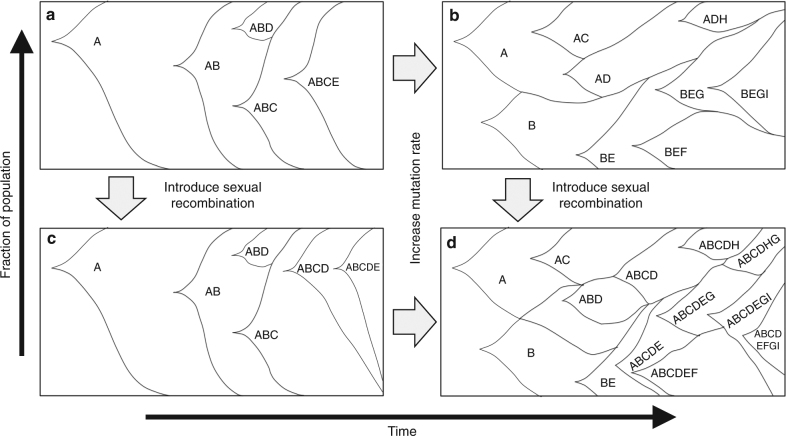
Theoretical impacts of mutation rate and sexual recombination on population structures. **a** Mutationally limited fitness landscape, where most mutations fix in a single sweep with very little inter-clonal competition. **b** Increased mutation rate over (**a**), more competition and faster evolution observed, but some mutations are lost due to inter-clonal competition. **c** Adding sexual recombination to (**a**), due to few available beneficial mutations, no strong influence on evolution is observed. **d** Both sexual recombination and increase in mutation rate from (**a**), more rapid evolution is observed and fewer beneficial mutations are lost due to clonal interference

The use of adaptive laboratory evolution (ALE) for phenotype development relies on the principles of Darwinian evolution, wherein individuals with mutations conveying a fitness benefit can be selected for, and enriched. As an asexual bacterial population evolves and accumulates mutations in a given environment, mutations imparting beneficial phenotypes expand as a subpopulation; the introduction of sexual recombination^1–6^ and an increased mutation rate^7–13^ have both been shown to facilitate swifter adaptation in certain environments (fitness landscapes). However, how mutation rate and sexual recombination impact the evolutionary trajectory of a population along the fitness landscape of a given environment has not been fully explored experimentally. In addition, the impact of mutations and their interactions (epistasis) on the fitness of a cell, outside a few well-characterized examples in specific environments, is limited^1,7,9–16^. Existing studies on microbial evolution has yielded significant insights into the dynamics of evolving populations^1,8,16–18^. Because the availability, frequency, and strength of beneficial and deleterious mutations vary depending on the environment, ALE in different environments can yield different intra-population dynamics. In many environments, clonal interference shapes population structure during microbial adaptive evolution, wherein many beneficial mutations co-arise and compete as subpopulations (Fig. 1)^19,20^. The presence of sexual recombination is theorized to have two advantages for an evolving population: reducing clonal interference thereby speeding evolution (Fig. 1d) and to break apart hitchhiking (deleterious or neutral) mutations from strong beneficial mutations. Additionally, an increased mutation rate is expected to expedite the rate of fitness improvement by increasing the availability of beneficial mutations (Fig. 1c), but also increase the accumulation of deleterious mutations. Recent work has explored the relationships between sexual recombination and mutation rate on microbial adaptive evolution^4^ and further elucidated the impact of sexual recombination on the molecular dynamics during evolution^21^. However, given the rarity of sexual recombination in simpler and faster growing organisms, the advantages of sexual recombination, especially over a range of mutation rates, is significantly less clear^4^.

Here we aim to determine the influences of both sexual recombination and mutation rate on evolving populations of *Escherichia coli* in well studied fitness landscapes. Specifically, we aim to determine the impacts of these two parameters on the speed of adaptation in fitness landscapes of varying complexities, and the effects of sexual recombination on population structure. We harness a previously developed “genderless” strain of *E. coli*, capable of sexual recombination through the F-plasmid conjugation machinery. Using this system, we previously demonstrated that in situ sexual recombination can increase the rate of adaptation during ALE experiments in complex fitness landscapes^3^. In this work, we introduced an inducible mutator system into the genderless strain. The resulting strain, capable of sexual recombination and with a modulatable mutation rate, was evolved in parallel with an asexual counterpart in three different environments. Each of these backgrounds has been selected to represent different expected fitness landscapes allowing us to determine when sexual recombination and mutation rate provide an advantage to evolving populations. From the evolution experiments in each fitness landscape, we find that increased mutation rate expedite adaptation in some environments tested, and that sexual recombination further speed up adaptive evolution in some environments. We also observe strong evidence of an in situ recombination event that help to alleviate clonal interference and generate a superior genotype in a sexual *E. coli*.

## Results

### Evolutions with chloramphenicol and trimethoprim challenge

Two antibiotics were chosen as two of the challenges for evolution, both with glycerol as the carbon source. Previous work on *E. coli* adapting to chloramphenicol (CM) challenge provides qualitative evidence of a smooth adaptation rate indicative of the presence of many smaller beneficial mutations^7^; and we have previously demonstrated that sexual strains acquired antibiotic resistance more rapidly when evolved in the presence of CM. Therefore, the adaptive landscape for CM resistance is likely complex, and adaptation to CM is expected to significantly benefit from sexual recombination and slightly benefit from increased mutation rate. Trimethoprim (TM) targets the DHFR protein responsible for folic acid biosynthesis^22^. Previous work has identified that TM tolerance occurs primarily through large fitness sweeps from sequential mutations in *folA* ^23^. Not surprisingly, our previous work showed that the use of the genderless strain had no benefit to evolving populations of *E. coli* subjected to TM challenge^3^. Thus, the TM adaptive landscape is less complex and only an increased mutation rate is expected to enhance the rate of adaptation to TM.

We compared the strains with or without sexual recombination each with or without induction of the mutator phenotype using the following four strains/conditions: genderless with arabinose induction (genderless ara+), genderless without arabinose induction (genderless ara−), asexual with arabinose induction (asexual ara+), and asexual without induction (asexual ara−). In each evolution, all populations were maintained in exponential growth via serial batch transfer during the mid-log phase; consequently, the population fitness is ascribed by the growth rate during the exponential phase with minimal influence from the duration of growth lag or cell survival during the stationary phase. An exponential ramping scheme for the antibiotic challenges was implemented to maintain the evolving populations in the presence of an antibiotic concentration consistent with the true drug tolerance of each population^3,7^. For each strain with or without arabinose induction, six replicate populations were evolved in a glycerol minimal media supplemented with either CM or TM. Each population was first pre-adapted to glycerol without antibiotic supplementation for four serial transfers.

**Fig. 2:**
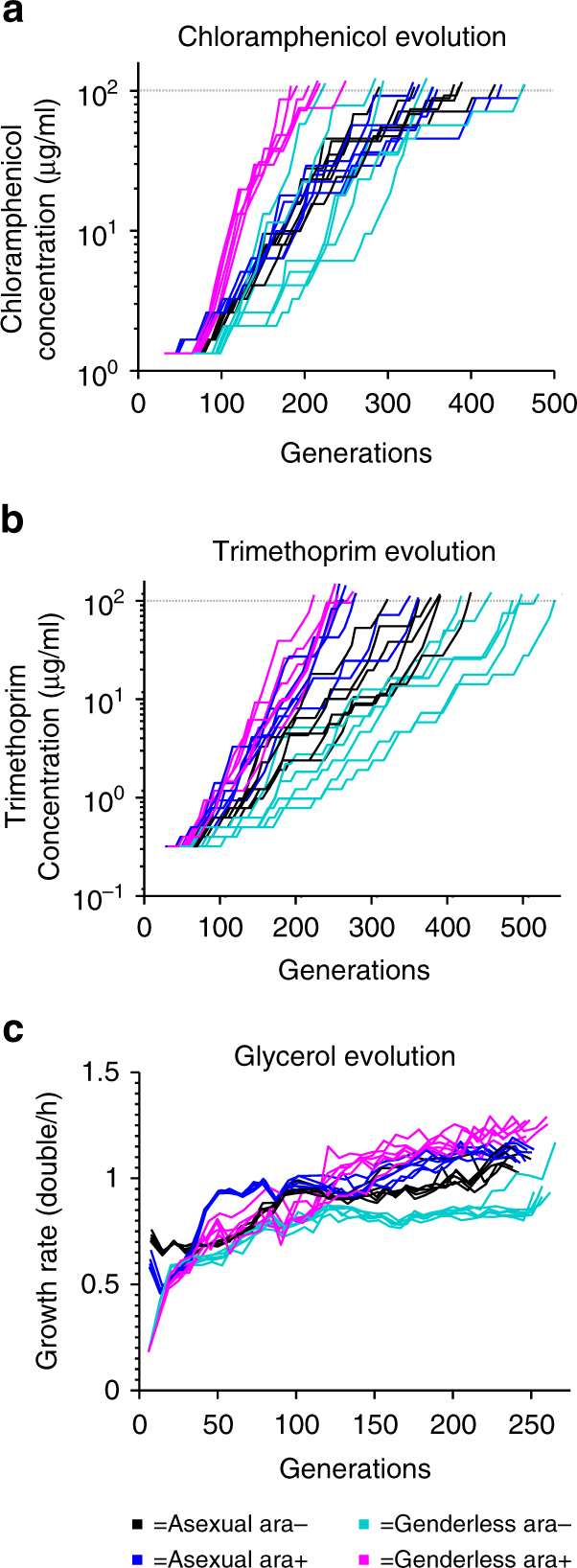
Fitness improvements during ALE. Black: asexual ara− populations. Blue: asexual ara+ populations. Turquoise: genderless ara− populations. Magenta: genderless ara+ populations. **a** Chloramphenicol resistance vs. generations in G9+CM evolution. **b** Trimethoprim resistance vs. generations in G9+TM evolution. **c** Growth rate vs. generations in G9 evolution. The dotted line in **a** and **b** represent the chosen threshold antibiotic resistance level

Using a target CM tolerance of 100 µg/mL, a significantly more rapid improvement in tolerance was observed in the genderless ara+ populations relative to both the asexual ara+ or ara− populations (Mann–Whitney *U* test, <0.01) and the genderless ara− population during evolution (Mann–Whitney *U* test, <0.01) (Fig. 2a). The genderless ara− populations displayed a similar rapid improvement in tolerance as the genderless ara+ populations, but this was preceded by prolonged periods of no improvement. The asexual ara+ and ara− populations had a similar rate of improvement.

We quantified the evolutionary data by fitting the antibiotic tolerance vs. the number of generations to a two-term exponential, with one fitted term for rate of exponential improvement “*a*” and the other for time lag before improvement “*b*”, *C*_*ab*_ = *C*_*abo*_ e ^(*a*(*t*+*b*))^. The value of the fitted term “*b*” for the genderless ara− populations is much higher than the asexual strains (Mann–Whitney *U* test, <0.05) (Supplementary Fig. 1). The genderless populations have larger “a” term (Mann–Whitney *U* test, <0.05) (Supplementary Fig. 1). The results suggest that sexual recombination facilitates a more swift increase in resistance, probably by combining compatible determinants of CM tolerance. The increased mutation rate in genderless ara+ populations likely provided an increased supply of mutations for generating beneficial recombinants, thereby reducing the number of generations before improvement in tolerance was observed.

Similar to CM evolution, the four strains/conditions were evolved in TM with a target tolerance of 100 µg/mL (Fig. 2b). The fitted lag, “*b*”, parameter of the ara+ populations showed more rapid tolerance development over their ara− counterparts (Mann–Whitney U test, <0.05) (Supplementary Fig. 1). The sexual ara+ population has a slightly, but not statistically significantly, faster increase in fitness. As expected, sexual recombination has little advantage in TM fitness landscape, but an increased mutation rate is advantageous.

### Evolution for faster growth rate on glycerol

*E. coli* is not well adapted to glycerol as a primary carbon source; therefore, during adaptive evolution to TM and CM, the populations may have also been evolving for growth on glycerol. Thus, the third evolution was performed without antibiotic challenge, solely for improved growth rate on glycerol. The Palsson lab has identified consistent evolutionary trajectories for more rapid growth on glycerol, wherein a few larger leaps in a relative fitness of ~30–80% were reproducibly observed from mutations in *glpK* and *rpoB/C* ^24^. Mutations in *glpK*, which encodes for glycerol kinase, have been linked to altering three primary functions leading to better growth: auto-regulation, catabolite repression, and enzyme activity^24–26^. Mutations observed in *rpoB/C* likely lead to complex global regulatory changes^24^. Correspondingly, as *E. coli* evolve toward faster growth on glycerol minimal media, we expect populations to benefit most from increased mutation rate; sexual recombination should provide little benefit when there are few, very strong, beneficial determinants. Additionally, we expect all of the evolved populations to settle on a similar final fitness after both strong mutations fix.

Each strain in both induced and uninduced conditions were also evolved for faster growth on glycerol to assess the impacts of sexual recombination and mutation rate on rates of adaptation on this carbon source. Both the genderless ara− and genderless ara+ populations exhibited lower initial fitnesses on glycerol compared with their asexual counterparts, possibly due to a physiological adaptation period caused by the metabolic burden of the conjugation machinery^27^; a shorter physiological adaptation period from LB to glycerol is also observed in the asexual populations. It takes approximately three serial transfers for the genderless strain to physiologically adapt to the glycerol minimal media. This metabolic transition period is reproducibly observable if populations grown on glycerol media are transferred to rich media and then back to glycerol media (data not shown). Therefore, relative fitness was calculated using the specific growth rates with the third transfer as baseline. The ara+ populations reached fitness plateaus more rapidly than their ara− counterparts (Fig. 2c). In the genderless populations, the genderless ara+ reached the first plateau in fitness at generation ~50, whereas the genderless ara− reached a similar fitness later, at generation 80. The genderless ara+ reached another plateau at generation ~130, but the genderless ara− never reached this fitness peak by the termination of the experiment. A similar trend was observed between the asexual populations, the asexual ara+ reached fitness plateaus at generations 60 and 150, while the asexual ara− reached similar plateaus much later, at generations 90 and 225.

**Fig. 3:**
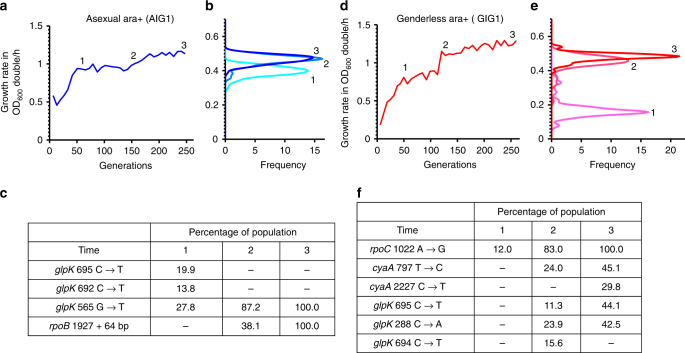
Correlating changes in population average fitness with dynamics of mutations in AIG1 and GIG1. **a** Population fitness data for population AIG1 (asexual ara+). **b** Population fitness distribution at Time = 1, 2, and 3 for AIG1. **c** Population-level sequencing data for observed mutations at Time = 1, 2, and 3 for AIG1. **d** Population fitness of GIG1 (genderless ara+). **e** Population fitness distribution at Time = 1, 2, and 3 for GIG1. **f** Population-level sequencing data for observed mutations at Time = 1, 2, and 3 for GIG1. See Supplementary Fig. 2 for correlation between population fitness and average isolate fitness

Prior studies have determined that mutations in *rpoB/C* and *glpK* are primarily responsible for *E. coli* adaptation on glycerol^24–26^ Assuming the fitness landscape of *E. coli* on glycerol is more simple, as in the case with TM, we would expect the benefit of sexual recombination to be minimal. Thus, it was unexpected that the genderless ara+ populations had a higher final growth rate than the asexual ara+ (Student's *t*-test, *p*-value <10^−4^). It is possible that the genderless strain can allow for the best combination of co-arising *rpoB/C* and *glpK* mutations to be generated, and could allow for weaker beneficial mutations to be preserved through mutational sweeps, avoiding clonal interference and leading to better adaptation. On the other hand, sexual recombination can also generate recombinants with higher relative fitness, which can potentially reduce the fitness diversity by rapidly outcompeting less fit genotypes. To assess these possibilities, we assayed the fitness diversity in the final populations; if diversity of beneficial mutations is preserved in the genderless populations, we would expect the final populations to have a larger distribution of fitnesses relative to the asexual ara+ populations. The measured growth rate distributions correlate well (qualitatively) with the average growth rates of the final evolved populations (Fig. 3 and Supplementary Fig. 2). However, no significant differences in the variances of the genderless and asexual ara+ populations were observed (F-test >0.05).

As fitness distributions do not definitively provide information regarding the preservation of beneficial mutations in the population, we sequenced population samples at three time points from evolving populations genderless ara+ 1 and 2 (GIG1 and GIG2, respectively), and asexual ara+ 1 and 2 (AIG1 and AIG2, respectively). Each time point chosen corresponded to an increase in population fitness to a fitness plateau as well as the final population. Each population contained 1–10 mutations that were detected at a statistically significant level, and in all sequenced populations (genderless and asexual), mutations in both *glpK*, and *rpoB*/*rpoC* were observed (Supplementary Table 1). This was not unexpected, as the Palsson group identified mutations in *glpK* in 47/50 independent ALE experiments^25^; the *glpk288* and *glpK692* mutations we identified in our evolved populations have been previously observed^25^. Interestingly, mutations in *cyaA* (adenylate cyclase) were observed only in the two genderless populations. Adenylate cyclase is responsible for production of cAMP and is crucial for alternative carbon source utilization. Mutations in *cyaA* have not been previously reported in glycerol-evolved *E. coli*, but have been observed in lactate-evolved *E. coli* ^28^. The remaining, extraneous mutations identified have not been previously observed to be beneficial for and had no obvious connection to growth rate on glycerol, and will not be analyzed in detail in this work. To connect the dynamics of the evolution to the sequencing data, at each sequenced time point, we measured the fitness distributions of the populations (see Figs. 3a and d for a representative asexual and genderless population).

The population average fitness of AIG1 exhibited a large increase between generation 60 (Time = 1) and generation 150 (Time = 2), with no further increase between Time = 2 and generation 250 (Time = 3), as depicted in Fig. 3a–c. Population-level sequencing data revealed three different mutations in *glpK* at Time = 1. Prior to Time = 2, a mutant containing the *glpK565* mutation likely acquired a mutation in *rpoB*, which expanded as a subpopulation to reach 100% frequency. No additional mutations were detected in this population by the end of the evolution experiment. Therefore, it is not surprising that the population fitness was largely unchanged between Time = 2 and Time = 3, as the superior genotype (containing both *glpK565* and *rpoB1927*) outcompeted all other less fit mutations and swept the population. On the other hand, the genderless populations retained more heterogeneity (see Fig. 3f for population GIG1 as an example). In population GIG1, the first observed expansion was a mutation in *rpoC1022*, which reached ~12% frequency by generation 50 (Time = 1), and 100% fixation by generation 250 (Time = 3). At generation 130 (Time = 2), several additional mutations (one in *cyaA* and three in *glpK*) reached significant frequencies, drastically increasing the population fitness. Contrary to the asexual ara+ population, where a single pair of mutations fixed, in the genderless ara+ population, a diversity of mutations was maintained until the termination of the experiment as the population fitness increased.

### Evidence of an in situ sexual recombination event

**Fig. 4:**
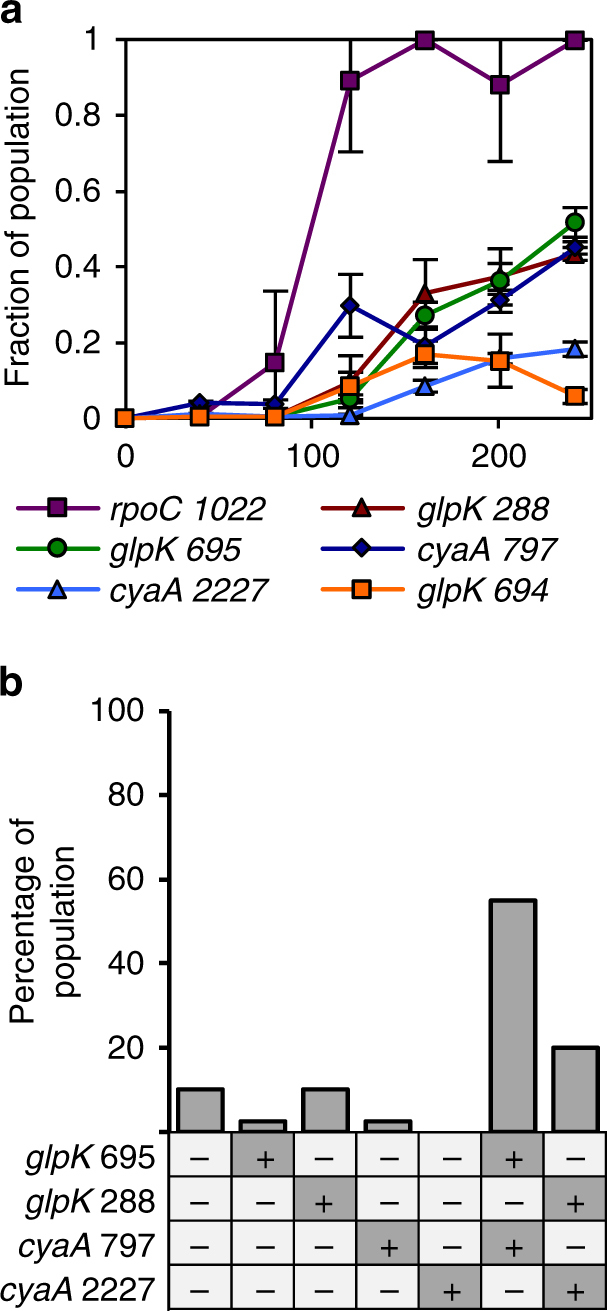
Dynamics of select mutations for population GIG1. **a** Changes in mutation frequencies in the population over time. Error bars are s.d. (*n* = 3 technical replicates). **b** Genotype distribution of 100 random isolates in the final population tested with allele specific PCR. Dark gray box with a “+”: mutated genotype. White box with a “−”: wild-type genotype

As the genderless population is expected to recombine mutations from independent clones in the population, we aimed to determine if recombination indeed occurred between different subpopulations. First, six mutations identified in GIG1 were tracked by allele specific PCR (Fig. 4a), mutations previously excluded as extraneous were ignored for this analysis. The results confirmed the *rpoC1022* mutation fixes in the population and *glpK694* is largely lost by Time = 3. The combined frequencies of the remaining four mutations, *glpK288* and *glpK695* and *cyaA2227* and *cyaA797* were confirmed to be well above 100%, indicating some of these four mutations must coexist as pairs of mutations in a single genotype. To identify the different combinations that are present in the population, we randomly picked 96 isolates from the Time = 3 population and used allele specific PCR to determine the combinations of these four mutations in the colony isolates (Fig. 4b). Of the surveyed mutations, in addition to the mutation in *rpoC*, most of the isolates contain either a single mutation (*glpK288*, *glpK694*, or *cyaA797*) or a pair of mutations (either *glpK695+cyaA797* or *glpK288+cyaA2227*). Notably, individual colonies with mutations in either *glpK695* or *cyaA797* and isolates with both of these two mutations were identified; these data suggest that either a sexual recombination event occurred between the subpopulations with individual mutations in *glpK695* and *cyaA797*, to generate recombinant with both mutations, or the *glpK695* mutation spontaneously arose in a mutant with the *cyaA797* mutation or vice versa. Given the low probability of an identical mutation occurring spontaneously in more than one subpopulation, it is more probable that the *glpK695*+*cyaA797* mutant arose from a recombination event.

### Ascertaining the fitness landscape

**Fig. 5:**
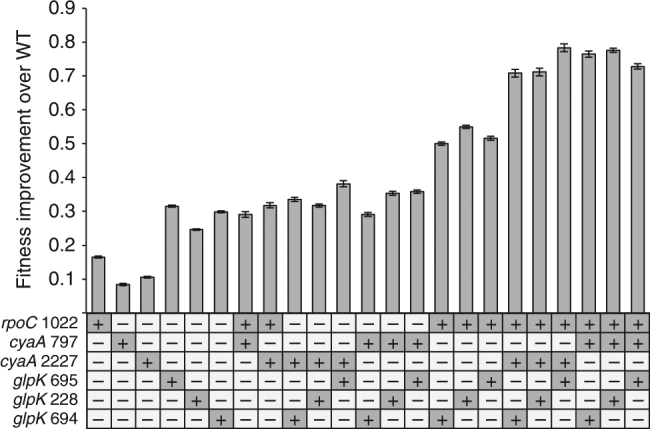
The relative fitness improvement over the wild type of each reconstructed mutant. Dark gray box with a “+”: mutated genotype. White box with a “−”: wild-type genotype. Error bars are s.e.m. (*n* = 36 biological replicates for single mutant and wild type and *n* = 18 biological replicates for double and triple mutants)

**Fig. 6:**
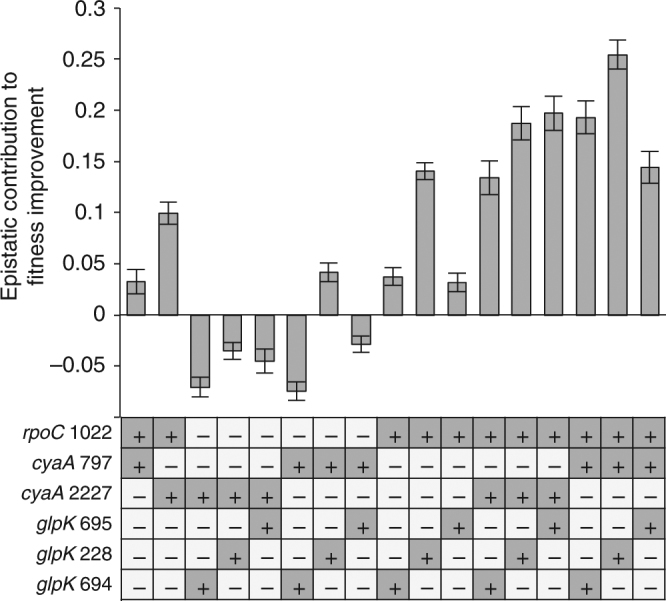
Epistatic interactions between mutations. Each bar depicts the epistatic contribution to fitness improvement for each reconstructed genotype with multiple mutations, calculated relative to the sum of component single mutations. Dark gray box with a “+”: mutated genotype. White box with a “−”: wild-type genotype. Error bars are s.e.m.

To determine the accessible fitness landscape of this particular genderless ara+ population, we reconstructed each and every possible combination (except for combinations of mutations within the same gene) of the three mutated genes (*rpoC, glpK*, and *cyaA*) into the wild-type BW25113. The growth rates of each single, double, and triple mutant constructs were compared relative to the wild type on G9 media. As shown in Fig. 5, all of the single mutants exhibited a statistically significantly improved growth rate compared to the wild-type (*t-*test, <10^−3^), showing that all mutations identified in *rpoC*, *glpK*, and *cyaA* are beneficial, though the *glpK* mutations conferred the highest fitness benefit followed by *rpoC* then the *cyaA* mutations. The double mutants exhibited greater growth rates than their single mutant counterparts (*t-*test, <10^−3^), suggesting reciprocal sign epistasis is not present; except for when *cyaA2227* was combined with *glpK694* and when *cyaA797* was combined with *glpK695*, where no additional increase in fitness were observed beyond that of the single *glpK* mutation (Supplementary Tables 2 and 3). All triple mutants exhibited fitness values that are statistically significantly improved over their possible double mutant counterparts (*t-*test, <0.02). Epistatic interactions between different mutations were quantified by comparing the fitness of each double or triple mutants against the sum of its component individual mutations. All combinations of mutations with an *rpoC1022* mutation yielded positive epistasis, whereas combinations without *rpoC1022* yielded variable epistasis (Fig. 6). Of special note, the *rpoC1022*+*glpk228* combination yielded strong positive epistasis. Likewise, all of the triple mutants, which all have the *rpoC1022* mutation, displayed strong positive epistasis. To confirm that the beneficial mutations and epistatic interactions identified in this work are not specific to the BW25113 background, we also reconstructed these mutations in MG1655. The results showed similar fitness trends as those observed in BW25113 (Supplementary Fig. 4).

### Use of the genderless system for rapid recombinant formation

The combinations of three mutations we observed in the adaptive evolution, *rpoC1022* with either *glpK695*+*cyaA797* or *glpK228*+*cyaA2227*, represent just two of the peaks on the fitness landscape, but not necessarily the local maxima as illustrated in Fig. 5, which depicts the fitnesses of all possible combinations. To gain further insight into the potential benefit of sexual recombination to simultaneously generate and select for beneficial combinations of mutations, we reconstructed the six individual mutations identified from the adaptive evolution in the genderless strain and used them to seed a short selection experiment on glycerol. After eight transfers (55 generations of selection), each population had several mutations that reached moderate frequencies (though the *rpoC* mutation is consistently observed). The sum of the frequencies of all mutations in each population was ~2, indicating the average number of mutations in each cell in our final populations was ~2, strongly suggesting that recombination occurred (Table 1).

**Table 1:** Fraction of each seed mutation in each of six replicate populations after 55 generations of selection

Replicate	Mutation fraction in population					
	1	2	3	4	5	6
*rpoC1022*	0.911 ± 0.039	0.839 ± 0.055	0.868 ± 0.025	0.936 ± 0.019	0.821 ± 0.011	0.805 ± 0.019
*cyaA797*	0.003 ± 0.001	0.002 ± 0.001	0.001 ± 0.001	0.001 ± 0.001	0.007 ± 0.002	0.003 ± 0.001
*cyaA2227*	0.498 ± 0.056	0.181 ± 0.018	0.252 ± 0.028	0.326 ± 0.056	0.455 ± 0.031	0.088 ± 0.008
*glpK695*	0.007 ± 0.001	0.019 ± 0.006	0.015 ± 0.004	0.010 ± 0.005	0.155 ± 0.056	0.018 ± 0.006
*glpK288*	0.399 ± 0.106	0.672 ± 0.022	0.832 ± 0.012	0.592 ± 0.063	0.367 ± 0.019	0.840 ± 0.008
*glpK694*	0.426 ± 0.055	0.260 ± 0.073	0.109 ± 0.034	0.495 ± 0.097	0.504 ± 0.060	0.151 ± 0.025
Total	2.244 ± 0.137	1.978 ± 0.096	2.077 ± 0.052	2.360 ± 0.130	2.309 ± 0.090	1.905 ± 0.034

Errors in s.d. (*n* = 3 technical replicates)

## Discussion

The overall objective of this work was to determine how sexual recombination and increased mutation rate can influence adaptation in various fitness landscapes, for use as a tool for ALE. The previously developed genderless strain, capable of continuous in situ sexual recombination, was further modified with an inducible mutator phenotype for this work. The combination of both sexual recombination and an increased mutation rate was beneficial in expediting adaptive evolution in all fitness landscapes tested. The genderless ara+ populations reached the target tolerance for CM ~40.7% more rapidly, the target tolerance for TM ~16.8% faster (although not statistically significant), and achieved a ~13.6% higher growth rate on glycerol minimal media than the next best performing strain/condition. The results strongly suggest that the combination of sexual recombination and an increased mutation rate is capable of enhancing ALE in microbial systems in any fitness landscape.

In all antibiotic challenged evolutions, populations without an increased mutation rate were observed to have extended lag before phenotype improvement; this is indicative of mutationally limited regimes where the mutational supply is too low for sexual recombination to be beneficial. This was especially evident with the CM challenge, where the genderless ara- population improved more quickly than both asexual ara+ and ara− populations but was subjected to an extended delay. Interestingly, while increased mutation rate was expected to be beneficial in CM challenged evolution, the asexual ara+ populations did not perform better than the asexual ara−. We conjecture that increased mutation rate is only marginally beneficial in this case and with additional replicates, the asexual ara+ and ara− may become statistically separable.

Next-generation sequencing of the glycerol-evolved populations consistently identified the expected *rpoB/C* and *glpK* mutations in all sequenced populations, and was able to provide strong insight into the different intra-population dynamics occurring upon the introduction of sexual recombination; specifically, we uncovered strong evidence that sexual recombination alleviated clonal interference (see further discussion below). While some of our assumptions hinge on similarity of BW25113 and MG1655, because the growth advantages of each mutation/combination of mutations was consistent, we are confident the adaptive landscape of BW25113 is similar to MG1655 and the same conclusions drawn by Palsson and co-workers on the fitness landscape of glycerol are applicable here^24–26^. Population GIG1 maintained multiple different combinations of mutations in various subpopulations including those of lesser fitness benefit (*rpoC1022*+*cyaA797* 31% improvement, vs. *rpoC1022* with either *glpK288* or *glpK695*, 51% and 54% improvement respectively [fitness differences *t*-test, <10^−4^]), and eventually produced a pair of triple mutant subpopulations from these. On the other hand, in the asexual population, all the lesser-fit mutations besides the two that fixed were lost.

With advances in NGS, recent work has started to experimentally demonstrate the benefits of sexual recombination in alleviating clonal interference and enhancing adaptation. In work by McDonald et al.^21^ the ability of yeast to purge hitchhikers was demonstrated using sequence-level dynamics. Additionally in a recent work by Chu et al.^29^ using the genderless and an HFR control strains we had previously developed, a recombination event during in situ adaptive evolution was observed for the first time; however, the relative fitness impacts of the individual mutations and recombinants were not determined, thus the exact benefits of recombination were not fully assessed. In this work, we identified a specific case of a sexual recombination event that likely helped to maintain a weaker beneficial mutation (mutations in *cyaA*) in the sexual population. Mutations in *cyaA* were only observed in the genderless and not the asexual populations. Thus, we hypothesized that maintenance of beneficial mutations in *cyaA* in the sexual populations is due to recombination between *cyaA* mutants with other beneficial mutations to generate an overall more fit genotype. Analysis of population GIG1 provided evidence supporting this hypothesis. In population GIG1, subpopulations containing either *rpoC1022*+*cyaA797* or *rpoC1022*+*glpK695* and a separate subpopulation containing *rpoC1022*+*glpK695*+*cyaA797* were identified in the same population sample, providing strong evidence of a recombination event occurring between *rpoC1022*+*cyaA797* and *rpoC1022*+*glpK695*. However, this observation may also be explained by very rare mutational events in which an identical mutation occurred twice in different subpopulations within the same evolution, with the occurrence of a recombination event being the more probable explanation. To mathematically weigh the two hypotheses, we compared the frequency of generating the desired mutant in a population composing of 5% genotype A and 5% genotype B, conditions similar to those observed at generation 125 in the evolving population (Fig. 4a). We had previously developed a model to estimate recombinant formation and propagation of the genderless strain in a batch culture^30^. We modified the parameters of our previously developed model to better simulate the experimental conditions (population size, mutant fitness, etc.) of this work, and compared the expected number of double mutants formed from recombination events to the expected number of spontaneous double mutants (see Methods). Results showed that the chance of the double mutant arising from mating is ~6 orders of magnitude more frequent than from spontaneous mutation (~10^6^ vs. ~10^−0.5^, respectively, using mutation frequency of 1/100 per cell per generation).

If recombination events are sufficiently frequent, it is surprising we did not observe all other combinations of mutations at Time = 3. Upon termination of the experiment, the GIG1 population had settled primarily on two of the four possible combinations of *glpK* and *cyaA* mutations. We hypothesize that two factors inhibited the establishment of the other possible genotypes, and the loss of one *cyaA* mutation by Time 3. First, the heights of peaks along the fitness landscape appear to be similar, as multiple combinations of three beneficial mutations yielded similar relative fitness values that are significantly higher than that of the double mutants. The improvement from the fittest double mutant to the least fit triple mutant is ~7.1% (the average improvement from double to triple mutant is ~17%); however, between triple mutants (the highest peaks on the fitness landscape) the largest difference in fitness is only ~4.3%. Accordingly, any recombinants harboring any combination of the three mutated genes established initially would rapidly expand, limiting the opportunity for alternate triple mutants to form. Second, we posit the distance between the two genes of interest *glpK* and *cyaA*, of about ~100 kb, is small enough to dampen the frequency of generating recombinants. Our prediction is further supported by the results of the selection experiment using the reconstructed genderless single mutants, where the results from six independent populations revealed the generation and enrichment of several random combinations of mutations, indicating underlying stochasticity. If recombination was more frequent, such that all possible combinations can be generated rapidly, we would have expected a more consistent mutational profile in each population.

In conclusion, we first demonstrated that mutation rate and sexual recombination have differential impacts on the speed of adaptation depending on the fitness landscape. An increased mutation rate was able to provide an advantage in environments where limited beneficial determinants are present, and sexual recombination was similarly able to improve ALE in environments where beneficial determinants are weaker and more frequent. When combined, the benefits of both led to the observed near universal improvement in ALE. Focusing on the mutator populations evolved for growth on glycerol, we further found the population structure to be more heterogeneous in the presence of sexual recombination; more beneficial mutations were maintained in the sexual populations as would be expected based on the theorized impacts of both sexual recombination and increased mutation rates on population structure shown in Fig. 1d. Additionally, we observed strong evidence of sexual recombination acting to alleviate clonal interference through a recombination event that combined beneficial mutations from separate subpopulations to found a more fit population with both mutations. Finally, we demonstrated that using reconstructed genderless single mutants, beneficial recombinants can be generated more rapidly, with applications in strain construction.

## Methods

### Strain construction

Overexpression of *dam* has been shown to increase mutation rate by reducing the time a cell has to harness methyl-directed mismatch repair^31^. Disruptions in the mismatch repair system and possibly loss-of-function allele of *dam* (to a smaller degree) have been shown to increase frequency of recombination^32,33^; thus we chose to use an overexpression of *dam* to minimize the impact of the mutator phenotype on recombination frequency. Conditional mutator versions of the previously developed genderless strain and asexual control^3^ were constructed to allow modulation of mutation rate. All strains used in this work are listed in Supplementary Table 4. The genderless strain contains a chromosomally integrated F-plasmid, with the surface exclusion genes *traST* knocked out. This allows effective in situ sexual recombination; for detailed characterization see Winkler et al.^3^ and Peabody et al.^30^. The *dam* gene was inserted behind the arabinose promoter of PLA2^34^ and integrated into the chromosome at the λ phage attachment site of the previously constructed HFR-2xoriT-SFX (genderless) and BW25113 2xoriT strains^3^ to generate the conditional mutator strains, HFR-2xoriT-SFX-P_bad_ *dam* (sexual) “genderless” and the BW25113 2xoriT-P_bad_ *dam* (asexual) strains. Both strains are ara−, ∆(araD-araB)567. The mutation rates of each strain were determined by standard fluctuation tests^35,36^; when induced with 3.33 µM arabinose, the mutation rate is increased in both the genderless and asexual control strains on glycerol minimal media (G9) by 22.69-fold (Upper CI and 32.64 Lower CI 13.98) and 17.73-fold (Upper CI and 26.77 Lower CI 9.94), respectively. G9 is identical to M9 minimal media except glucose is replaced with 5% (v/v) glycerol as the carbon source and supplemented with 50 mg/L tryptophan. Additionally, we confirmed the genderless strain maintained the ability to mate when grown on glycerol minimal media (data not shown).

### Glycerol evolution

Six single colonies for each genotype (genderless and asexual) were grown up in LB media overnight. An appropriate dilution from each culture was used to inoculate ~10^7^ cells into two test tubes, one test tube containing 5 mL of glycerol minimal media (G9) the other with 5 mL of G9 media with 3.33 µM arabinose, grown at 37 °C and 275 rpm. The cultures were then propagated into fresh, pre-warmed media when the OD_600_ value approached approximately 0.5 (~7 generations) to maintain the cells in log phase growth. For each serial transfer, an initial OD_600_ of ~0.004 was used. Samples collected every other serial transfer were cryo-preserved at −80 °C in 15% glycerol.

### CM and TM evolutions

The antibiotic challenged evolutions were performed similarly as in the glycerol evolution with the following exceptions. After the first four serial transfers, the cultures were propagated with antibiotic challenge (starting concentration 1.33 μg/mL CM or 0.32 μg/mL TM). If a culture exhibited growth rate greater than 0.583 doubles/hour (7 generations in 12 h) the antibiotic concentration was increased by 25%, otherwise the culture was transferred to fresh media with the same antibiotic concentration. If a culture exhibited improvements in antibiotic resistance for three consecutive transfers, the concentration was ramped up by 50% instead of 25% for all subsequent transfers until a transfer with no improvement was observed.

### Exponential fitting

Exponential rate of improvement and lag were estimated for the two antibiotic evolutions, using the ft function in MATLAB with parameters for ramp rate, *a*, and lag time, *b*, in *C*_*ab*_ = *C*_*abo*_ e ^(*a*(*t*+*b*))^.

### Sequencing

Population samples were grown up from 20 μl frozen stock on LB media; isolates were selected from colonies of the population streaked onto LB plates then grown up on LB. Genomic DNA was extraction from either single isolates or population samples using ZR Fungal/Bacterial gDNA miniPrep (Zymo Research). NGS library preparations and sequencing were performed by the Texas A&M Institute for Genome Sciences and Society core facility at Texas A&M University using 75 bp single-end reads on the Illumina NextSeq 500. Five hundred million reads passed quality control with an average coverage for each population sample of ~500 reads/bp. Fastq files are available from the NCBI SRA (accession no. SRP110624). The sequencing data were aligned to *E. coli* BW25113 reference^37^ with breseq^38^. The lowest frequency of a mutation discussed in this work was detected at a frequency of 7.6%.

### Allele frequency tracking

Genomic DNA was extracted as above. qRT-PCR was performed on the genomic DNA using the DyNAmo HS SYBR Green qPCR kit following the manufacturer’s instructions, using both mutant and wild-type primers for each mutation with three technical replicates each (see Supplementary Table 5 for primer sequences)^39,40^. The allele specific primers were validated by constructing a standard curve with different percentages of mutant and wild-type gDNA (Supplementary Fig. 5).

### Genotype tracking

Frozen stock of GIG1 Time = 3 population was diluted and plated on LB. Allele specific PCR was performed on individual isolates to determine the presence of mutations *cyaA2227, cyaA797*, *glpK695*, and *glpK288*. Colonies were selected and resuspended in 50 μl of sterile water. The colony suspension was tested for each mutation using both wild type and mutant allele specific primers. Mutations were determined from the resultant band distribution. One isolate of each identified genotype was further verified with Sanger sequencing.

### Population fitness measurements

Ninety-six randomly chosen colonies of the population of interest were inoculated into G9 media for growth for 24 h. Two microliters of the culture was transferred to a 96-well microtiter plate containing 98 μl of G9 media. Plate reader with shaking and incubation capabilities (TECAN Infinite M200) was used to track the growth in each well at 37 °C with 270 rpm orbital shaking. To eliminate edge effects, we excluded the edges of the 96-well microtiter plates.

### Strain reconstruction

We reconstructed several mutations identified in GIG1 Time = 3 population in wild-type backgrounds using the procedure outlined by Datsenko and Wanner and the *cat*-*sacB* selection-counterselection cassette^41,42^. The selection was performed on LB plates with 12 μg/mL CM and the counter selection was performed on LB plates with no NaCl and 6% (w/v) sucrose (See Supplementary Table 5 for primer sequences). All mutations constructed in the BW25113 background were verified with Sanger sequencing.

### Strain fitness measurements

The fitness of reconstructed strains was measured using the same method used for the population isolates. Epistasis was estimated using an additive model^43–46^, where the net epistasis e_i,j,k_ for a given combination of mutations in cyaA (i), glpk (j), and rpoC (k) was estimated with:

$$e_{i,j,k} = \mathop {\sum }\limits_{i,j,k} \frac{{a_{i,j,k} - \left( {a_im_i + a_jm_j + a_km_k} \right)}}{{\left( {a_im_i + a_jm_j + a_km_k} \right)}}$$

where *m*_*n*_ is 1 if the mutation was present and 0 if the mutation was absent and *a*_*n*_ is the fitness of the associated mutation (or combination of mutations).

### Selection experiment with genderless mutant constructs

Single mutants of the genderless strain were cultured overnight in LB from individual colonies. Five microliters of the overnight culture was normalized and used to inoculate fresh cultures in 5 mL of G9 in test tubes and cultured for 5 h at 37 °C and 275 rpm. Each individual culture was normalized to OD_600_ ~1 and pooled. Nineteen microliters of the pooled culture was inoculated into fresh G9 according to the protocol for the glycerol evolution outlined above for six serial transfers.

### Estimate of mating vs. spontaneous mutation rate

Input to the previously developed model of recombination frequency (see ref. ^30^) were the following parameters: Genotype A = 5%, Genotype B = 5%, Genotype C = 90% Genotype AB = 0%, P_i_ = 1.6 × 10^6^; P_f_ was taken to be when the population reached 2 × 10^8^ cells and *μ*_AB_ = 1.8*μ_o_, *μ*_A_ = 1.55*μ_o_, *μ*_b_ = 1.55*μ_o_, *μ*_c_ = 1.2*μ_o_. The result of one transfer with the above parameters is that at P_f_, AB = 0.0012% (2 × 10^5^ cells per transfer). For the mutation frequency estimate, the same starting population size, fitnesses, and compositions as above were used. The generations were assumed to be stepwise with a mutant frequency per generation of (population size)×(mutation frequency, 1/100)×(1/[mutational space genome size × 4]). Mutant frequencies from each generation were propagated with a fitness of twice the WT until the end of growth (2 × 10^8^ cells). The result of one transfer with the above parameters is 0.78 expected mutants per transfer.

### Data availability

Sequencing data have been deposited in the NCBI Sequence Read Archive (SRA) with accession code SRP110624. Other data generated or analyzed during this study are available in this published article and its Supplementary Information files, or from the corresponding author upon request.

## Electronic supplementary material

Supplementary Information
