# Supplementary material for: Sexual recombination and increased mutation rate expedite evolution of Escherichia coli in varied fitness landscapes
Source: Nat Commun. 2017 Dec 13;8:2112. doi: 10.1038/s41467-017-02323-4 (PMC5727395; doi:10.1038/s41467-017-02323-4)
Supplement: Supplementary file 1 — Supplementary Information [file 41467_2017_2323_MOESM1_ESM.pdf]

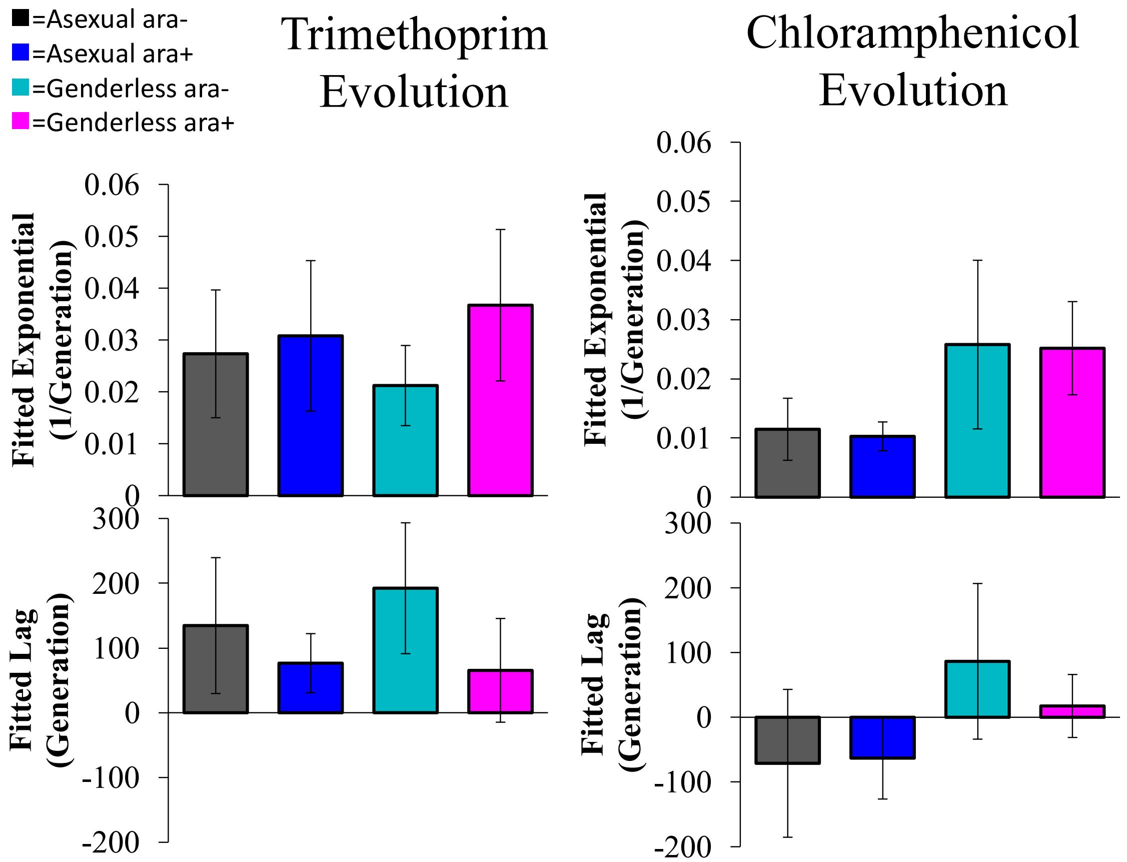

Supplementary Figure 1: Fitted parameters, "a" and "b", for the TM and CM evolutions. On the top is the average "a", the fitted exponential term, for the 6 replicate populations of each strain/condition pairing. On the bottom is "b", the fitted lag term. Errors bars are one s.d. (n = 6 independently evolved populations each).

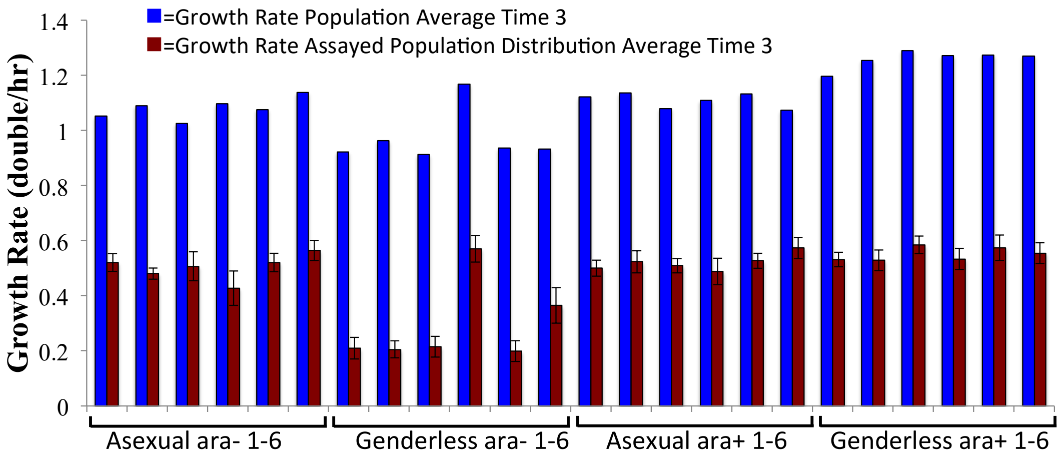

Supplementary Figure 2: The growth rates measured at Time = 3 during the evolution (blue) are compared to the fitness distributions measured in the populations at Time = 3 (red). Population fitness data was based on 96 randomly chosen colonies. Errors bars are one s.d. (n = 96 independent colonies).

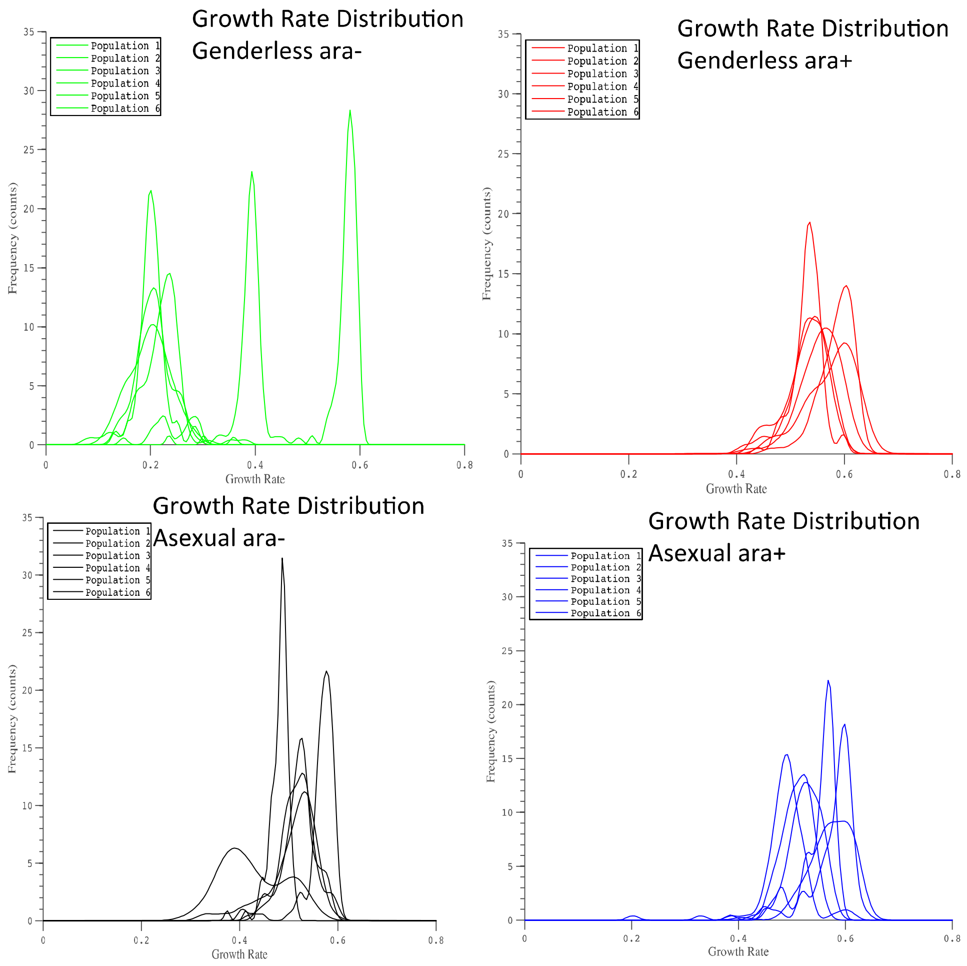

Supplementary Figure 3: Growth rate distributions of each set of evolved populations at Time = 3 for the growth rate on glycerol evolution. Distribution based on 96 randomly chosen colonies.

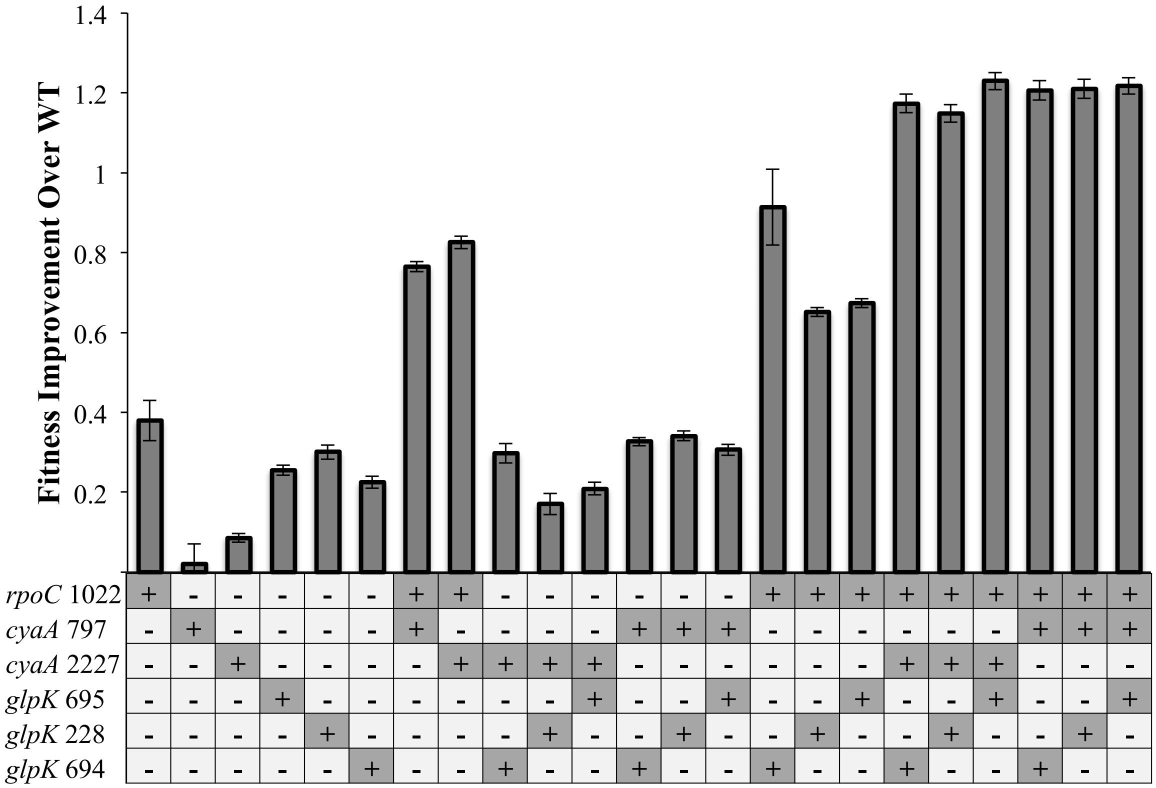

Supplementary Figure 4: The relative fitness improvement over the wild-type of each reconstructed mutant for strain MG1655. Dark gray box with a '+': mutated genotype. White box with a '-': wild-type genotype. Error bars are s.e.m. (n = 15 biological replicates for single mutant and wild-type and n = 9 biological replicates for double and n = 6 biological replicates for triple mutants).

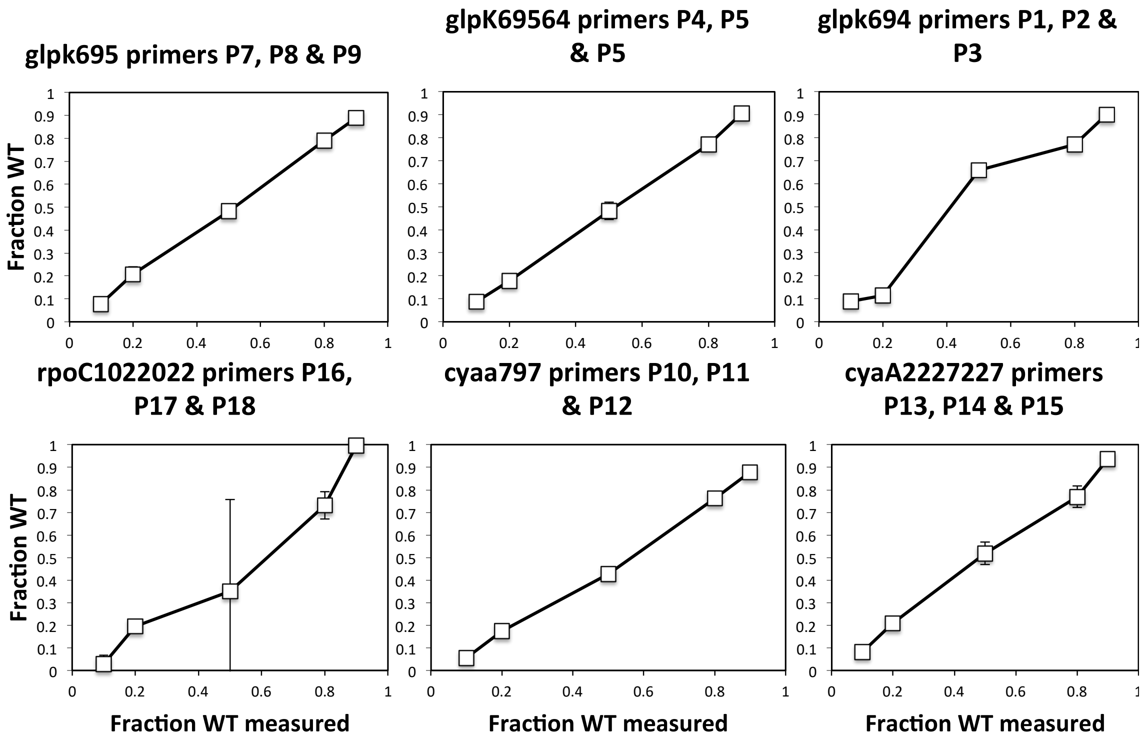

Supplementary Figure 5: Calibration curves for each set of primers used to measure allele frequency, comparing measured DNA composition (wild type to mutant) versus true composition, errors bars are one s.d. (n = 3 technical replicates each).

Supplementary Table 1: Curated mutation list from each sequenced population and time.

Population	Gene	Location	Mutation	Frequency
GIG1 Time 1	*curA*	519	A→C	11.70%
GIG1 Time 1	*rpoC*	1022	A→G	12.00%
GIG1 Time 1	*leuQ*	80	.→G	11.60%
GIG1 Time 2	*mraZ*	238	C→T	12.60%
GIG1 Time 2	*glsA*	829	G→A	10.90%
GIG1 Time 2	*cyaA*	797	T→C	24.00%
GIG1 Time 2	*glpK*	695	C→T	11.30%
GIG1 Time 2	*glpK*	694	C→T	15.60%
GIG1 Time 2	*glpK*	288	C→A	23.90%
GIG1 Time 2	*azoR1*	150-161	-12 bp	7.50%
GIG1 Time 2	*rpoC*	1022	A→G	83.00%
GIG1 Time 3	*lpxC/secM*	+91/-140	G→T	12.80%
GIG1 Time 3	*cof/ybaO*	+34/-119	T→.	10.60%
GIG1 Time 3	*ubiF/glnX*	+153/+1	G→T	10.30%
GIG1 Time 3	*cyaA*	797	T→C	45.10%
GIG1 Time 3	*cyaA*	2227	C→T	29.80%
GIG1 Time 3	*glpK*	695	C→T	44.10%
GIG1 Time 3	*glpK*	288	C→A	42.50%
GIG1 Time 3	*azoR1*	150-161	-12 bp	26.30%
GIG1 Time 3	*rpoC*	1022	A→G	100.00%
GIG2 Time 1	*ygfA/traM*	-220/-194	A→C	11.00%
GIG2 Time 1	*traJ*	124	.→T	10.80%
GIG2 Time 1	*ccmB*	149	G→A	14.90%
GIG2 Time 1	*pdxK*	315	G→A	25.00%
GIG2 Time 1	*gspB*	202	C→T	29.00%
GIG2 Time 1	*rpoC*	1022	A→G	25.70%
GIG2 Time 2	*miaB*	1306	G→A	11.70%
GIG2 Time 2	*treA*	1372	A→G	11.60%
GIG2 Time 2	*rsxC*	2012	C→A	10.00%
GIG2 Time 2	*gspB*	202	C→T	10.60%
GIG2 Time 2	*cyaA*	1378	A→G	19.50%
GIG2 Time 2	*glpK*	196	C→T	27.10%
GIG2 Time 2	*glpK*	164	G→A	53.00%
GIG2 Time 2	*rpoC*	1022	A→G	72.00%
GIG2 Time 2	*traJ*	126	.→T	59.30%
GIG2 Time 2	*rpoC*	1401	-18 bp	13.40%

GIG2 Time 2	*hrpB/mrcB*	+85/-111	T→G	10.80%
AIG1 Time 1	*bcsQ*	218	A→C	29.80%
AIG1 Time 1	*glpK*	694	C→T	18.10%
AIG1 Time 1	*glpK*	164	G→A	11.20%
AIG1 Time 2	*yhjY*	6	G→T	11.30%
AIG1 Time 2	*glpK*	238	A→G	88.40%
AIG1 Time 2	*rpoB*	1330	G→T	10.80%
AIG1 Time 2	*rpoB*	1576	C→T	77.60%
AIG1 Time 3	*glpK*	238	A→G	100.00%
AIG1 Time 3	*rpoB*	1576	C→T	100.00%
AIG2 Time 1	*sseA/sseB*	+371/+447	G→T	15.50%
AIG2 Time 1	*glpK*	695	C→T	19.90%
AIG2 Time 1	*glpK*	692	C→T	13.80%
AIG2 Time 1	*glpK*	565	G→T	27.80%
AIG2 Time 2	*glpK*	565	G→T	87.20%
AIG2 Time 2	*rpoB*	1927	+64 bp	37.70%
AIG2 Time 3	*glpK*	565	G→T	100.00%
AIG2 Time 3	*rpoB*	1927	+64 bp	100.00%

Supplementary Table 2: p-values for comparisons of growth rates between single mutant constructs.

	Wild Type	*rpoC1022*	*cyaA797*	*cyaA2227*	*glpK695*	*glpK288*	*glpK694*
Wild Type	1	-	-	-	-	-	-
rpoC1022	2.15E-07	1	-	-	-	-	-
cyaA797	0.0107	0.000720	1	-	-	-	-
cyaA2227	0.000199	0.0445	0.135	1	-	-	-
glpK695	9.25E-19	4.75E-08	4.45E-13	1.85E-10	1	-	-
glpK288	1.17E-15	0.000234	5.63E-10	3.10E-07	2.13E-06	1	-
glpK694	1.61E-17	7.17E-07	1.93E-12	1.16E-09	0.195	0.00210	1

Supplementary Table 3: p-values for comparisons of growth rates between double/triple mutant constructs and their composite mutations.

Genotype	WT	rpoC	cyaA	glpK	rpoC+cyaA	rpoC+glpK	cyaA+glpK
rpoC1022 cyaA2227	4.49E-10	7.23E-05	1.11E-06	-	-	-	-
rpoC1022 cyaA797	6.22E-09	0.000178	3.59E-07	-	-	-	-
cyaA2227 glpK694	8.66E-14	-	6.01E-11	0.0472	-	-	-
cyaA2227 glpK288	1.46E-14	-	4.23E-08	0.00121	-	-	-
cyaA2227 glpK695	1.48E-07	-	5.42E-06	0.114	-	-	-
cyaA797 glpK694	1.70E-10	-	7.70E-06	0.452	-	-	-
cyaA797 glpK288	2.39E-11	-	7.42E-08	0.00173	-	-	-
cyaA797 glpK695	1.94E-16	-	1.11E-09	0.0261	-	-	-
rpoC1022 glpK694	8.45E-19	6.613-14	-	2.42E-09	-	-	-
rpoC1022 glpK288	2.65E-21	9.23E-17	-	7.638E-18	-	-	-
rpoC1022 glpK695	2.04E-18	3.89E-14	-	3.83E-09	-	-	-
rpoC1022 cyaA2227 glpK694	1.34E-11	3.54E-10	1.10E-10	2.54E-08	1.86E-07	0.000383	5.97E-07
rpoC1022 cyaA2227 glpK288	9.00E-11	2.05E-09	8.03E-10	2.34E-08	9.94-07	0.0159	1.16E-06
rpoC1022 cyaA2227 glpK695	6.861E-11	4.06E-10	3.97E-10	3.11E-08	1.48E-07	0.000566	8.12E-06
rpoC1022 cyaA797 glpK694	7.5229E-13	2.22E-11	1.07E-12	1.10E-09	2.10E-08	2.84E-05	1.27E-09
rpoC1022 cyaA797 glpK288	1.29E-19	4.70E-12	8.24E-14	2.08E-15	3.06E-10	1.03E-06	1.28E-11
rpoC1022 cyaA797 glpK695	6.97E-15	1.00E-11	2.00E-13	1.38E-10	8.20E-09	0.000190	9.76E-09

Supplementary Table 4: Strains utilized in this study.

Strain	Source
MG1655	CGSC
genderless (BW25113 HFR-2xoriT-SFX)	Winkler et al 2012[1]
BW25113 2xoriT	Winkler et al 2012[1]
genderless λ::[Pbad-*dam kan*]	this work
BW25113 2xoriT λ::[Pbad-*dam kan*]	this work
BW25113 *rpoC1022*	this work
BW25113 *cyaA797*	this work
BW25113 *cyaA2227*	this work
BW25113 *glpK695*	this work
BW25113 *glpK228*	this work
BW25113 *glpK694*	this work
BW25113 *cyaA797 glpK695*	this work
BW25113 *cyaA797 glpK228*	this work
BW25113 *cyaA797 glpK694*	this work
BW25113 *cyaA2227 glpK695*	this work
BW25113 *cyaA2227 glpK228*	this work
BW25113 *cyaA2227 glpK694*	this work
BW25113 *rpoC1022 cyaA797*	this work
BW25113 *rpoC1022 cyaA2227*	this work
BW25113 *rpoC1022 glpK695*	this work
BW25113 *rpoC1022 glpK228*	this work
BW25113 *rpoC1022 glpK694*	this work
BW25113 *rpoC1022 cyaA797 glpK695*	this work
BW25113 *rpoC1022 cyaA797 glpK228*	this work
BW25113 *rpoC1022 cyaA797 glpK694*	this work
BW25113 *rpoC1022 cyaA2227 glpK695*	this work
BW25113 *rpoC1022 cyaA2227 glpK228*	this work
BW25113 *rpoC1022 cyaA2227 glpK694*	this work
genderless *rpoC1022*	this work
genderless *cyaA797*	this work
genderless *cyaA2227*	this work
genderless *glpK695*	this work
genderless *glpK228*	this work
genderless *glpK694*	this work
MG1655 *rpoC1022*	this work

MG1655 *cyaA797*	this work
MG1655 *cyaA2227*	this work
MG1655 *glpK695*	this work
MG1655 *glpK228*	this work
MG1655 *glpK694*	this work
MG1655 *cyaA797 glpK695*	this work
MG1655 c*yaA797 glpK228*	this work
MG1655 *cyaA797 glpK694*	this work
MG1655 *cyaA2227 glpK695*	this work
MG1655 *cyaA2227 glpK228*	this work
MG1655 *cyaA2227 glpK694*	this work
MG1655 *rpoC1022 cyaA797*	this work
MG1655 *rpoC1022 cyaA2227*	this work
MG1655 *rpoC1022 glpK695*	this work
MG1655 *rpoC1022 glpK228*	this work
MG1655 *rpoC1022 glpK694*	this work
MG1655 *rpoC1022 cyaA797 glpK695*	this work
MG1655 *rpoC1022 cyaA797 glpK228*	this work
MG1655 *rpoC1022 cyaA797 glpK694*	this work
MG1655 *rpoC1022 cyaA2227 glpK695*	this work
MG1655 *rpoC1022 cyaA2227 glpK228*	this work
MG1655 *rpoC1022 cyaA2227 glpK694*	this work

Supplementary Table 5: Primers utilized in this study.

Name	Sequence 5'->3'
P1	CCG AAG TAT ACG GTC AGA CTA ACA TTG CCG
P2	TTC CGA AGT ATA CGG TCA GAC TAA CAT TGC CA
P3	CTC GCC AGT GTT CAT CAG CAT AAA GCA G
P4	CCAG ACA ATG GCG TTA TAG ATA GGC
P5	GCC AGA CAA TGG CGT TAT AGA TAG GA
P6	CTC CAC GCT GGT AGA AGT GCT G
P7	TAC GCG TGC CGC CTG TGC
P8	GAA TAC GCG TGC CGC CTG TGT
P9	CCG ATT ACA CCA ACG CCT CTC GTA C
P10	CCC CAT ACA AAG CGG TAC TGA AAA AAC T
P11	CCC CAT ACA AAG CGG TAC TGA AAA AAC C
P12	CAC GGC TGA GCT TTT CGC AC
P13	TTT GCC AGC GAA GGG ATC TTC C
P14	TTT GCC AGC GAA GGG ATC TTC T
P15	GTT GAT GAA GCT TGA GCC GTA GG
P16	GGT AAA CAG GGT CGT TTC CGT CTG AA
P17	GGT AAA CAG GGT CGT TTC CGT CTG AG
P18	GCG GAT AAC TTC GTC CAG GAT ATC C
P19	CGTAAAAACCCGCTTCGGCG
P20	ACATCCCGCAACCTGCGATT
P21	CCTCGCTGGGTGTGGAAGTC
P22	AGCAGGCGGGTGAAACAGTC
P23	CAT CGC TTC CAG CAC GTC AC
P24	GAT TGG TCT ACT GAT TGC GGT CAT TG
P25	GCGAAGATTGTGGCTCCACCCAGCATATACTGCTGCTTGACGAATTTTATATC AAA GGG AAA ACT GTC CAT AT
P26	CGGATAAGCCTCGCTTTCCGGCACGTTCATCACGAAAAATATTGCTGTAATGT GAC GGA AGA TCA CTT CG
P27	CAACGCATAGTTCACTTCGCCAGTCGGGCCGCAGGCGATGGTGGTCAGCAATC AAA GGG AAA ACT GTC CAT AT
P28	TTCAGAACAAAAAGCTTCGCTGTAATATGACTACGGGACAATTAAACATGTGT GAC GGA AGA TCA CTT CG
P29	GGCAAGGATTCGATACTATTCCTGTGTAACTTTCTTAAGGAACGAGAATGATC AAA GGG AAA ACT GTC CAT AT
P30	TGGCGAAACCCACCTTAAGGTGGGTTTTGTTATTTTGAGGGCTGAGGAAGTGT GAC GGA AGA TCA CTT CG
P31	CAGTAGAATTCATG AAG AAA AAT CGC GCT TTT TTG
P32	CAC TTG AAT TCC ATC CGC TTC TCC TTG AGA ATT A
P33	TTGTCGGTGAACGCTCTCCT
P34	CACATTGATTATTTGCACGG
P35	CGTTCATCTTTCCCTGGT
P36	AGGATGCGTCATCGCCATTA
P37	GGCATCACGGCAATATAC
P38	TCTGGTCTGGTAGCAATG
P39	ACTTAACGGCTGACATGG
P40	ACGAGTATCGAGATGGCA

Supplementary References

1. Winkler, J. & Kao, K. C. Harnessing recombination to speed adaptive evolution in Escherichia coli. *Metab Eng* **14,** 487–495 (2012).
